# Epidemiology and management of parathyroid gland disorders in Spain over 15 years: A retrospective multicentre analysis

**DOI:** 10.1371/journal.pone.0230130

**Published:** 2020-03-10

**Authors:** Josep Darbà, Alicia Marsà

**Affiliations:** 1 Department of Economics, Universitat de Barcelona, Barcelona, Spain; 2 Department of Health Economics, BCN Health Economics & Outcomes Research S.L, Barcelona, Spain; Universidade Nova de Lisboa Instituto de Higiene e Medicina Tropical, PORTUGAL

## Abstract

Parathyroid gland disorders are rare conditions with an incidence that displays great variability among populations. Its direct influence in calcium homeostasis originates variable symptoms that affect bone remodelling among other processes. This study aimed to provide data on the epidemiology and characteristics of patients admitted with these disorders in Spain between 2003 and 2017, and to analyse disease management and direct medical costs. Medical records in which a disorder of the parathyroid gland was registered as the admission motive were extracted from a nationwide hospital-discharge database via the Spanish Ministry of Health. Records from 12,903 patients were obtained, with predominance of female patients (74.70%) and of admissions due to hyperparathyroidism (90.23%). The number of patients admitted per year increased over the study period along the incidence of these disorders. The year 2017 incidence of hyperparathyroidism was 2.95 per 10,000, 4.03 per 10,000 in females and 1.37 in males; the same year, the incidence of hypoparathyroidism was 0.17 per 10,000. Length of hospital stay was significantly extended in patients with hypoparathyroidism (7.16 days), admitted mostly due to emergencies. Heart failure was diagnosed in more than 20% of admissions in patients with secondary and tertiary hyperparathyroidism and hypoparathyroidism, while this last group displayed the highest levels of mineral metabolism disruption. Parathyroidectomy was performed in 78.95% of all admissions for primary hyperparathyroidism. The total annual direct medical cost parathyroid gland disorders has increased over the study period, due to the increase of the costs associated to hyperparathyroidism, whereas the cost per patient remained relatively stable, with an average of €3,748, €3,430 and €3,737 for patients with hyperparathyroidism, hypoparathyroidism and other disorders of the parathyroid gland, respectively. This study provides novel data to extend the scarce available knowledge on parathyroid gland disorders’ epidemiology and management in Spain.

## Introduction

The disorders of the parathyroid glands are heterogeneous rare conditions generally characterised by the misregulation of calcium homeostasis due alterations in the secretion of parathormone (PTH), which, in normal conditions, regulates serum calcium levels [1,2]. Primary hyperparathyroidism (hyperPT) is the most common of these disorders, defined by the finding of hypercalcaemia alongside an increased serum PTH concentration [3]. Its incidence appears to correlate with age, with the highest rates found after the 55 years of age, and it is 3–4 times more frequent in females than in males [4,5]. Its overall incidence displays great variability within populations, various studies worldwide estimate annual incidence rates of around 2–4 per 10,000 individuals, although other estimations have reached the 80–90 per 10,000, in all cases with great fluctuations over time [5–7]. The diagnosis of asymptomatic patients via serum calcium screening is not rare; yet, the principal symptoms of hyperPT arise from the hypercalcaemia and may include fatigue, mild depression, polyuria and augmented bone remodelling with an increased risk of fracture [8,9]. In terms of management, when present, life-threatening hypercalcaemia is treated; subsequently, symptomatic patients are generally subjected to parathyroidectomy [9]. For asymptomatic patients, specific guidelines must be consulted. Secondary hyperPT, frequently derived from chronic kidney disease (CKD), and tertiary hyperPT, that follows long-standing secondary hyperPT with hypercalcaemia, are rarer parathyroid gland disorders presenting symptoms of bone, organic and metabolic disorders [10,11]. These forms are primarily pharmacologically treated and may occasionally require parathyroid surgery [10,12].

On the other hand, hypoparathyroidism (hypoPT) is a less common parathyroid gland disorder, characterised by low calcium levels in the blood serum due to inadequately low parathyroid hormone (PTH) levels [13]. Its incidence is around 1–3 per 10,000 and it is in most cases associated to a previous intervention to the neck, including total thyroidectomy [14–16]. Its symptoms result from the underlying hypocalcaemia causing increased neuromuscular irritability and typically appear along an increased bone mineral density and reduced bone remodelling, all pharmacologically treated [15,17].

The scarce and variable existent epidemiologic data for parathyroid gland disorders represents a major limitation that hampers the analysis of the management and costs of these disorders, and the use of real-world data for the development of management and treatment guidelines. This study aimed to provide data on the characteristics of patients admitted with parathyroid gland disorders in secondary care centres in Spain, along with an analysis of disease incidence, management and direct medical costs in the country.

## Methods

### Data extraction

Admission records corresponding to patients admitted with a parathyroid gland disorder in secondary healthcare centres (hospitals and specialised care centres) in Spain between 2003 and 2017 were obtained from the Spanish discharge database for Hospitalisation and Specialised Care via the Spanish Ministry of Health.

The dataset is codified using the 9th revision of the International Statistical Classification of Diseases and Related Health Problems (ICD9) until 2015 and the 10^th^ revision (ICD10) after 2016. The records of patients with a parathyroid gland disorder registered as the admission motive were identified using the codes: 252.xx, E20.x and E21.x. The codes to identify the different classes of hyperPT were introduced in 2006. Extraction criteria claimed 13,897 files that corresponded to 12,903 single patients.

Files did not contain any parameters identifying healthcare centres and medical history, which are re-coded within the database maintaining records anonymised, in accordance with the principles of Good Clinical Practice and the Declaration of Helsinki. This research did not involve human participants and there was no access to identifying information; in this context the Spanish legislation does not require patient consent and ethics committee approval [18].

### Data analysis

The first admission per patient registered during the study period was used for patient classification and description. The direct medical costs of secondary care were calculated based on the standardised average expenses of admissions and medical procedures determined by the Spanish Ministry of Health, including all expenses related to the admission (examination, medication, surgery, costs associated to personnel, medical equipment and resources) and excluding the costs of prescription medication.

Descriptive values are presented using mean and standard deviation (SD) or data range. Incidence was estimated as the proportion of patients admitted with parathyroid gland disorders within the database, excluding the groups with an insufficient annual patient number to preserve data reliability. Odds ratio (OR) with 95% confidence interval (CI) were used to evaluate associations in the diagnosis of comorbidities as indicated. Two-tailed T-student or one-way analysis of variance were used as appropriate, with a p<0.05 considered statistically significant. Statistical analyses were performed using Microsoft Excel© Professional Plus 2010 (Microsoft Corporation, Redmond, WA, USA).

## Results

Between 2003 and 2017, 13,897 admissions, corresponding to 12,903 patients, were registered in the database with a disorder of the parathyroid gland registered as an admission motive. Most of the patients included in the study were admitted due to hyperparathyroidism (90.23%), and female patients were predominant across all groups (Table 1). Due to the late introduction of the codes corresponding to primary, secondary and tertiary hyperPT, a large number of patients were registered with an unspecified type of hyperPT.

**Table 1:** Characteristics of patients admitted with disorders of the parathyroid gland in hospitals and specialised care centres.

Patient characteristics	Admissions	Patients	% females	Age (SD)
Disorders of the parathyroid gland	13,897	12,903	74.70	59.03 (15.58)
Hyperparathyroidism	12,539	11,667	74.05	59.70 (14.70)
Primary	8,080	7,518	72.17	59.98 (14.58)
Secondary	175	155	75.48	59.39 (17.87)
Tertiary	226	201	79.10	53.99 (15.09)
Unspecified	4,058	3,793	77.43	59.47 (14.87)
Hypoparathyroidism	1,069	955	82.72	52.72 (21.87)
Other specified disorders of the parathyroid gland ^a^	230	224	74.11	52.01 (18.71)
Unspecified disorder of the parathyroid gland	59	57	75.44	54.96 (19.29)

^a^ Haemorrhage of the parathyroid gland or cyst of the parathyroid gland.

**Fig 1:**
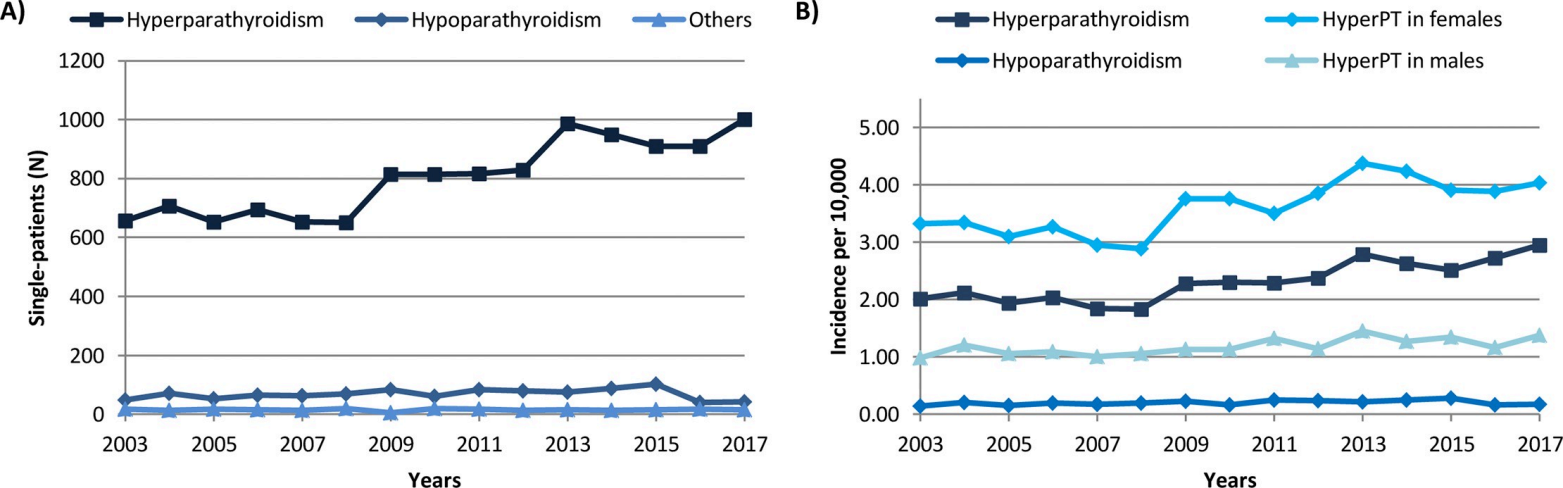
(A) Single-patients admitted with hyperparathyroidism (hyperPT), hypoparathyroidism (hypoPT) and other parathyroid gland disorders and (B) incidence of parathyroid gland disorders in Spain over time (2003–2017).

The annual number of single-patients admitted with hyperparathyroidism in healthcare centres in Spain increased over the study period (Fig 1A). Interestingly, patients’ mean age at admission increased over the years, from 59.02 years in 2003 to 61.07 years in 2017 in the group of patients with hyperPT (p<0.001), and remained stable in the group of patients with hypoPT.

The incidence of hyperparathyroidism was 2.33 per 10,000 individuals as calculated during the study period, whereas the incidence of hypoparathyroidism was 0.20 per 10,000 (Fig 1B). The year 2017 incidence of hyperPT was 2.95 per 10,000, 4.03 per 10,000 in females and 1.37 in males; the same year, the incidence of hypoPT was 0.17 per 10,000.

**Fig 2:**
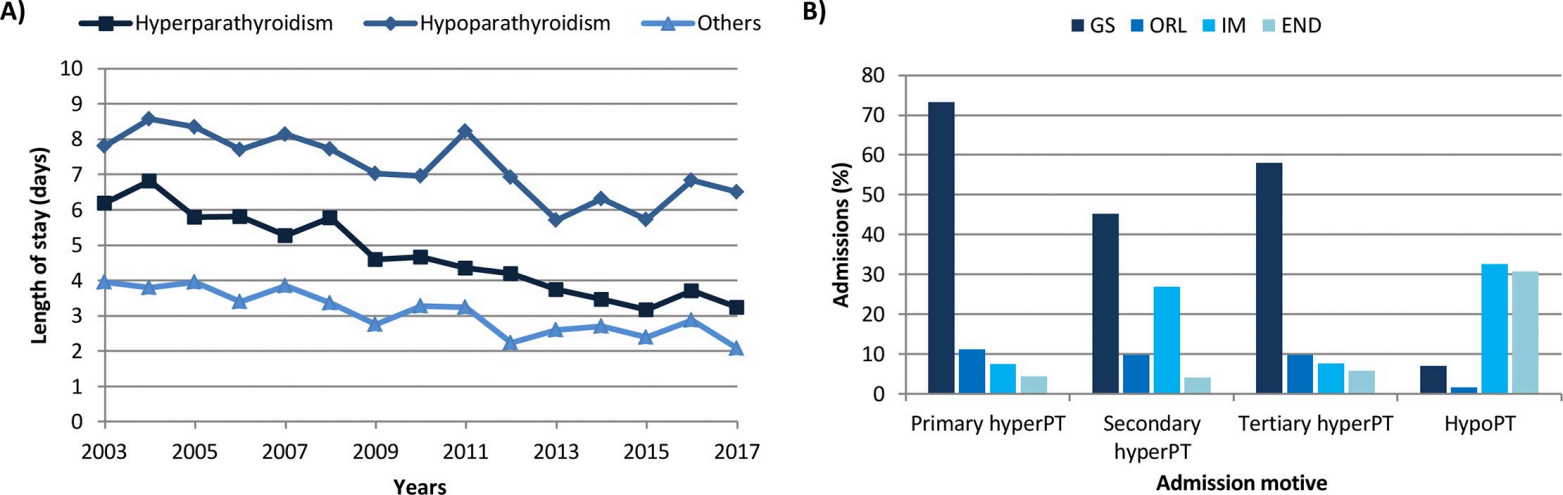
(A) Length of hospital stay over time and (B) principal services registered at discharge in patients with hyperparathyroidism (hyperPT) and hypoparathyroidism (hypoPT). GS, General Surgery; ORL, Otorhinolaryngology; IM, Internal Medicine; END, Endocrinology.

The vast majority of admissions due to hyperPT were scheduled (86.34%), whereas 85.69% of the admissions registered due to hypoPT were urgent and non-scheduled. Only 37 (0.27%) of all registered admissions were outpatient admissions, 34 due to hyperPT. Mean length of hospital stay (LOHS) was 4.57 days (0–144) for patients with hyperPT, and significantly prolonged in patients with hypoPT, averaging 7.16 days (0–72) (p<0.001). In addition, this parameter was found to decrease significantly over time from 6.19 days and 7.80 days in 2003 to 3.35 days and 6.50 days in 2017 for admissions due to hyperPT and hypoPT, respectively (p<0.001) (Fig 2A).

Patients with hyperPT were predominantly discharged by General Surgery services and those admitted with hypoPT were discharged by Internal Medicine and Endocrinology services (Fig 2B).

Secondary conditions registered on admission were analysed per subgroups of patients (Table 2). Various conditions were more frequently diagnosed in patients with hyperPT versus those with hypoPT, indicating the distinctive nature of these disorders, as essential hypertension (OR = 1.40, 95%CI 1.22–1.61), benign neoplasms of the parathyroid gland (OR = 30.71, 95%CI 12.74–74.03) and osteoporosis (OR = 5.09, 95%CI 3.29–7.87).

**Table 2:** Secondary conditions registered in more than 10% of all admissions of patients with parathyroid gland disorders.

Secondary diagnoses associated to disorders of the parathyroid gland	Admissions, %			
	Primary HyperPT N = 8,080	Secondary HyperPT N = 175	Tertiary HyperPT N = 226	HypoPT N = 1,069
Unspecified essential hypertension	33.66	26.29	34.96	23.57
Hypercholesterolaemia and hyperlipidaemia	18.99	20.57	26.55	16.09
Disorders of mineral metabolism	12.92	21.72	22.12	59.03
Hypercalcaemia	8.97	9.14	7.08	3.09
Hypocalcaemia	2.24	8.00	5.75	40.41
Magnesium	0.33	2.86	2.21	8.98
Benign neoplasm	21.18	18.86	7.96	2.53
Parathyroid gland	16.91	14.29	5.31	0.47
Heart failure	10.83	21.71	23.89	20.58
Atrial fibrillation	3.81	8.57	3.98	6.08
Diabetes	12.96	16.00	12.39	12.16
Osteoporosis	11.06	9.14	5.31	1.96
Hypertensive chronic kidney disease	3.95	15.43	16.81	4.40
Anaemia	3.04	12.00	7.52	10.01

The type of admission was associated with specific conditions; particularly, urgent admissions were associated with disorders of mineral metabolism (OR = 4.16, 95%CI 3.80–4.57), heart failure (OR = 1.81, 95%CI 1.63–2.00) and anaemia (OR = 3.11, 95%CI 2.62–3.70).

The medical procedures registered during the admission were analysed separately, revealing interesting information on patients’ condition and disease management (Table 3). In 69.11% of all admissions for hyperPT a parathyroidectomy was performed. Diagnostic imaging techniques were common in all patient groups, focusing on the parathyroid and thyroid glands and the analysis of bone density.

**Table 3:** Medical procedures registered during the admission of patients with parathyroid gland disorders.

Medical procedures registered during the admission	Admissions, %			
	Primary HyperPT N = 8,080	Secondary HyperPT N = 175	Tertiary HyperPT N = 226	HypoPT N = 1,069
**Surgical and therapeutic procedures**				
Parathyroidectomy	78.95	51.94	70.21	2.44
Thyroidectomy	12.92	6.20	14.36	0.61
Thyroid and parathyroid tissue reimplantation	1.39	3.88	13.30	0.92
Thymectomy and other excision of thymus	1.19	3.38	5.32	0.00
Injection or infusion of a therapeutic substance	6.03	8.53	8.51	19.13
Injection or infusion of electrolytes	4.37	6.98	3.72	6.10
Hemodialysis	0.42	3.10	7.45	0.71
**Diagnosis and imaging procedures**				
Diagnostic ultrasound	14.38	19.60	14.36	16.09
Head and neck	7.41	6.98	6.38	4.78
Biopsy of thyroid or parathyroid gland	6.06	3.88	5.32	0.92
Microscopic examination of blood	5.06	12.40	5.32	12.61
X-ray of various sites	4.89	17.05	4.79	12.72
Electrocardiogram	4.31	8.53	5.85	6.94
Parathyroid radioisotope scan	3.74	5.43	3.19	0.51
Thyroid scan and radioisotope function studies	3.32	4.65	2.13	0.20
Computerized axial tomography of head	3.16	6.20	3.19	11.29
Computerized axial tomography of abdomen	2.68	3.88	3.19	2.95
Computerized axial tomography of thorax	2.38	5.43	2.13	3.46
Bone radioisotope scan and density studies	2.04	4.65	1.06	1.02
Magnetic resonance imaging	1.38	5.43	2.66	4.07
Measurement of systemic arterial/venous blood gases	1.35	3.88	4.79	7.73
Microscopic examination of specimen from bladder and of urine	1.11	5.43	4.79	3.87

In a follow-up analysis focused on patients admitted for primary hyperPT, solely 15.50% of these patients were not subjected to parathyroidectomy during the study period. With a mean age of 64.31 (SD = 16.45), these patients were older than those with primary hyperPT that underwent parathyroid excision or resection (p<0.001), and diagnoses of hypercalcaemia (OR = 3.03, 95%CI 2.60–3.55) and heart failure (OR = 2.05, 95%CI 1.75–2.42) were increased in this group. In addition, 18.81% of these patients were subjected to a thyroidectomy.

**Fig 3:**
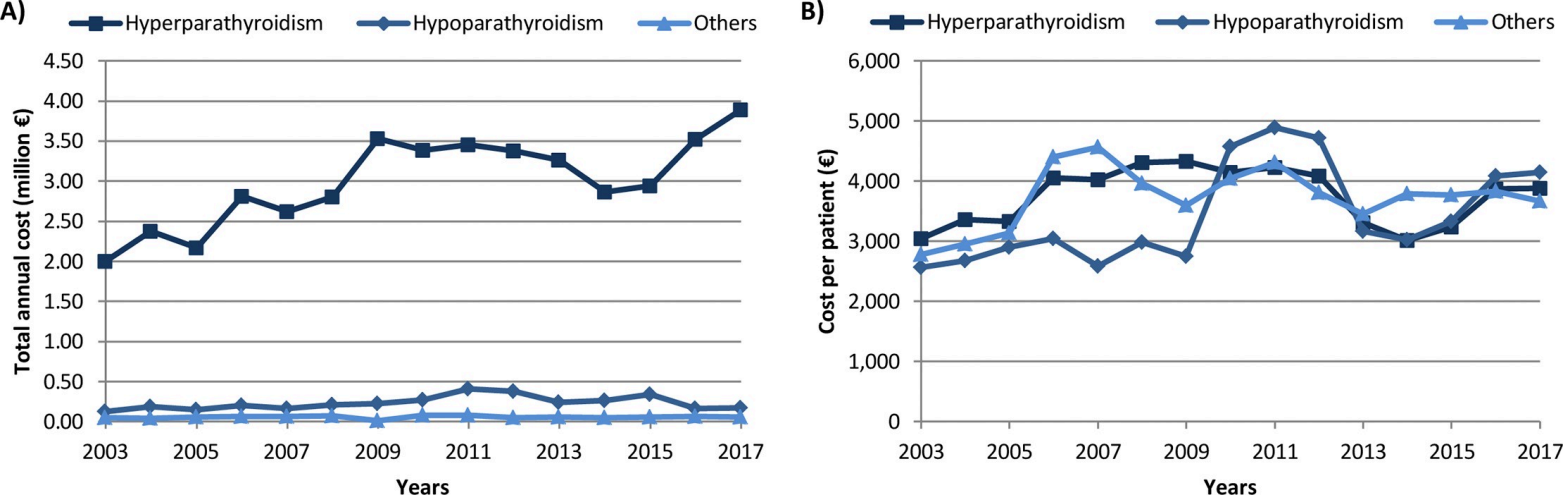
(A) Total annual direct medical cost of secondary care of parathyroid gland disorders (N = 12,903) and (B) annual direct medical cost per patient (2003–2017).

In terms of healthcare costs, the total annual direct medical cost parathyroid gland disorders has increased over the study period, due to the increase of the costs associated to hyperPT (Fig 3A), whereas no clear trend was identified in the cost per patient, due to groups with a small patient number and the big effects observed due to specific patients requiring intensive levels of care (Fig 3B). Nevertheless, data suggests a decrease in the cost per patient between 2011 and 2014.

The mean annual direct medical cost per patient with hyperPT was €3,748, €3,430 for those with hypoPT, and the annual cost per patient in those admitted with other disorders of the parathyroid gland was €3,737. Overall, the cost per admission increased significantly over the years, from a mean of €2,869, €2,510 and €2,779 in 2003 to €3,726, €3,666 and €3,668 in 2017 for patients with hyper PT, hypoPT and other disorders of the parathyroid gland, respectively (p<0.001).This cost was significantly higher in patients with hyperPT hospitalised for more than 50 days, reaching the €7,176 (p<0.001).

## Discussion

While few studies have explored the epidemiology and overall status of parathyroid gland disorders in Spain, data indicates an increase of their incidence within the Spanish population. Preliminary results obtained from a single centre in the north of Spain suggested an incidence rate of primary hyperPT that rapidly increased over the years and reached the 2.14 per 10,000 females between 2010 and 2014 [19]. The incidence of parathyroid gland disorders established in the present study, based on the evaluation of data obtained for the whole country, is not distant from those figures. The incidence of hyperPT also increased between 2003 and 2017, although data is not directly comparable. Similarly, estimations appear in line with measures obtained in the United States [5–7,9]. Such increasing tendencies could be correlated to an improvement in the diagnosis of these disorders.

Patients with parathyroid gland disorders admitted in hospitals and specialised care centres in Spain were mostly females and principally diagnosed with hyperPT. Interestingly, mean age was inferior in patients admitted with hypoPT (52.72 years) versus those with hyperPT (59.70 years). Further analysis described a group of patients that exhibited frequent diagnoses of essential hypertension, similarly to what was observed in the total hospitalised population the year 2015 (29.89%) [20]. In addition, in 16.91% of the admissions of patients with primary hyperPT underlying benign parathyroid tumours were registered, a percentage that is relatively low, as it was the diagnosis in-situ of hypercalcaemia. These conditions may not be registered in all of the admissions in previously diagnosed patients, in which clinicians may register hyperPT only; additionally, previous diagnoses obtained in primary care centres cannot be discarded. Contrarily, 40.41% of the patients with hypoPT were diagnosed with hypocalcaemia upon admission. In fact, exacerbated symptoms of hyper and hypocalcaemia, anaemia and heart failure were seemingly the reason for urgent admissions, registered principally in patients with hypoPT.

The rarity of these disorders has complicated its management and the standardisation of protocols. However, previous data indicates a slow implementation of international guidelines in the country, specifically, the application of the recommendations from the Third International Workshop for the management of primary hyperPT [21,22]. Further analyses of guideline adherence are not available for Spain; yet, its application cannot be discarded. In 2012, a consensus document was published by the Bone Metabolism Working Group of the Spanish Society of Endocrinology for the evaluation and follow-up of patients with normocalcemic primary hyperPT [23]. In parallel, international Workshop guidelines were updated in 2014 with recommendations to evaluate renal and skeletal involvement prior to parathyroid surgery in patients with primary hyperPT, while maintain the recommended age under 50 years [24]. The most recent guidelines for the management and treatment of primary hyperPT have been published by the National Institute for Health and Care Excellence of England and the PARAT program focusing on parathyroid gland disorders established by The European Society of Endocrinology [3,25]. Similarly, recommendations have been established for secondary and tertiary hyperPT and hypoPT [10,17].

Data herein suggests general guideline adherence, although this cannot be evaluated in detail. Diagnosis imaging techniques and blood examination were common in all patient groups. Almost 80% of the patients that were diagnosed with primary hyperPT were subjected to parathyroidectomy, leaving out a group of older patients with more frequent diagnoses of heart failure. The injection or infusion in-hospital of a therapeutic substance was increased in patients with hypoPT without further description.

On the other hand, internal Spanish dynamics may have influenced disease management. The decrease measured in the LOHS, coincides with a general decrease in this parameter described in the total hospitalised Spanish population over the past 20 years [20]. In addition, the annual increase of total medical costs correlates with the increase measured in the number of single patients admitted per year, with medical costs that only declined between 2011 and 2014, coinciding with a general decrease of hospitalisation costs in Spain due to budget adjustments during the economic crisis [20]. Data suggests an effect in the cost per patient that could not be measured directly due to the large variation measured among patients.

Finally, reference measures for the medical cost of parathyroid gland disorders are scarce. The year 2000, the estimated cost of minimally invasive parathyroidectomy for primary hyperPT was approximately €5,900, whereas the estimated cost associated to hypoPT was €5,036 in 2013, both measured in the United States [26,27]. Annual costs measured in the present study would be in line with such calculations, and the average €3,748, €3,430 and €3,737 estimated herein for patients with hyperPT, hypoPT and other disorders of the parathyroid gland, respectively, would represent a 2.3 fold increase on average versus the hospitalisation costs estimated for the total Spanish population [20].

Additional limitations may have influenced the results of this study. Only patients that have been admitted in hospitals and specialised care centres (inpatient and outpatient) are included in the study. This could introduce a bias, considering that, for some patients, diagnosis could be achieved in primary care centres. In addition, the database providing this data is codified with ICD9 and ICD10 codes, thus, data veracity is subjected to the accuracy of codification achieved within care centres. The codes identifying primary and secondary hyperPT were not fully used until 2006; its slow introduction and the large number of patients with an unspecified type of hyperPT impeded the estimation of incidence and any temporal analysis per each hyperPT type. Future evaluations in Spain and other European countries will be required to validate these results and to extend the analysis of parathyroid gland disorders epidemiology to other countries.

## Conclusions

Data describing the epidemiology and burden of disorders of the parathyroid gland is scarce, which hampers the formulation of clinical guidelines based on real-world data. This study describes patient profile, management and cost of these disorders in Spain, as well as its incidence, that continues to increase. Despite the relevance of primary hyperPT, the development and revision of management guidelines focusing on hypoPT patients appears necessary, considering the large percentage of patients requiring urgent care.
